# Oral Submucous Fibrosis in Pediatric Patients: A Systematic Review and Protocol for Management

**DOI:** 10.1155/2019/3497136

**Published:** 2019-04-01

**Authors:** Anuj Jain, Saumya Taneja

**Affiliations:** ^1^Department of Trauma and Emergency Medicine, All India Institute of Medical Sciences, Bhopal, Madhya Pradesh, India; ^2^Department of Dentistry, All India Institute of Medical Sciences, Bhopal, Madhya Pradesh, India

## Abstract

**Aim:**

To conduct a systematic review evaluating the cases of oral submucous fibrosis in pediatric patients.

**Material and Method:**

Systematic review was conducted using PRISMA guidelines. The article focused on oral submucous fibrosis in pediatric patients were included. A total of five manuscripts were included in our systematic review. The prevalence of OSMF in pediatric patients, gender distribution, causes, and clinical presentation were reviewed.

**Results:**

On systematically reviewing, a total of 10 cases of OSMF in pediatric patients were found. The youngest patient reported to be diagnosed with OSMF was of 2.5 years of age. Female preponderance was noticed. All the patients had the habit of areca nut chewing which subsequently led to fibrosis.

**Conclusion:**

Such a rapid increase in the rate of OSMF among pediatric population is a potential danger to the society. The habit of areca nut chewing is the major cause for this dreadful condition. Lack of health consciousness and low level of education are the major factors for initiation of this habit among children. Therefore it is imperative for the parents and school as well as government authorities to take serious actions.

## 1. Introduction

Oral submucous fibrosis (OSMF) is a chronic insidious disease that affects oral cavity, may also involve pharynx or esophagus, and may be associated with vesicle formation [1, 2]. This condition was first reported by Schwartz in 1952 and by Joshi in 1953 in India [3]. Its premalignant nature was described by Paymaster [4]. An incidence of carcinoma occurring from OSMF varies from 2 to 30% [5]. This premalignant condition occurs in Indian subcontinent predominantly with a prevalence rate of 0.5%, more commonly affecting females in a ratio of 3:1 [6, 7].

OSMF has an uncertain etiology. Various factors considered as probable causes of OSMF are capsaicin which is a component of chilli products, areca nut chewing, autoimmunity, hormonal factors, deficiency of nutritional factors like Vitamin B12 and iron, and genetic predisposition showing increased frequency of HLA 10, DR3, DR7 [8–11]. Areca nut is classified as Group I carcinogen by International Agency for Research on Cancer (IARC) [12]. Areca nut is the fourth most common social drug after nicotine, ethanol, and caffeine which have got deleterious effects on oral health [13]. The alkaloids, tannins, and nitrosamines present in areca nut are genotoxic and cytotoxic [14]. It is traditionally known as paan supari which is held in the buccal vestibule and chewed slowly over a period of time.

Clinical diagnosis of OSMF is based on its signs and symptoms which include paleness, burning sensation and ulceration of oral mucosa, recurrent stomatitis, xerostomia, and difficulty in swallowing or phonation along with occasional leukoplakia. It may be followed by fibrosis and stiffness of buccal mucosa and tongue which then leads to trismus and dysphagia [15, 16]. OSMF can also cause cardiac arrhythmia, asthma exacerbation, acute psychosis, and gut upset.

Performing a biopsy for OSMF is not recommended because it leads to further fibrous scar formation worsening the condition [8, 10]. Management of OSMF concentrates more on preventive aspect which includes counseling of the patients, refraining them from habits, and regular checkup. Other treatment modalities are injection of steroids, chymotrypsin or hyaluronidase, use of micronutrients, lycopene, CO_2_ laser, interferon gamma, turmeric, placental extracts, etc. In case of fibrosis, surgical interventions like myotomy, coronoidectomy, or excision of fibrous bands can be practiced. Procedures like insertion of stents, physiotherapy, local heat therapy, and mouth opening exercises are other alternatives that can also be attempted [17–21]

With more than 600 million people practicing the habit of areca nut chewing, cases of OSMF are increasing at an alarming rate. The most commonly affected group is 20-40-year-old adults [1]. Although occurrence of OSMF in pediatric patients is rare but cases of 7-15-year-old children have still been observed [15, 16]. More and more cases of OSMF among children are being documented lately because of easy availability, low cost, and sweet taste of areca nut. Therefore we conducted this systematic review to understand the prevalence and nature of pediatric OSMF and its social causes along with the prevention and treatment that can be initiated to protect our younger generation.

## 2. Methodology

### 2.1. Search Strategies

Systematic review was conducted using PRISMA guidelines. An exhaustive literature search was done using electronic databases like Medline, Cochrane database, and Google Scholar by two investigators. Search terms used were ‘oral submucous fibrosis' and ‘children' using Boolean operators AND. The relevant manuscripts published only in English language were given full consideration. Additional manual search was performed by reviewing the references of selected articles. Grey literature was also evaluated for unpublished manuscripts but no data was found. Titles and abstracts of the selected articles were studied and evaluated for inclusion in the systematic review.

### 2.2. Inclusion and Exclusion Criteria

The studies on oral submucous fibrosis in pediatric patients (first decade) were included in the review. Studies on any other premalignant condition or the ones involving adults were excluded. The manuscripts in any language apart from English, cases above 10 years, those with doubtful diagnosis, and incomplete information were also excluded.

### 2.3. Data Extraction and Quality Assessment

After evaluating the titles, keywords, abstracts, full articles, and their references and applying inclusion as well as exclusion criteria, a total of five manuscripts were selected for inclusion in our systematic review. The prevalence of OSMF in pediatric patients, gender distribution, causes, and clinical presentation were reviewed.

## 3. Results

**Figure 1:**
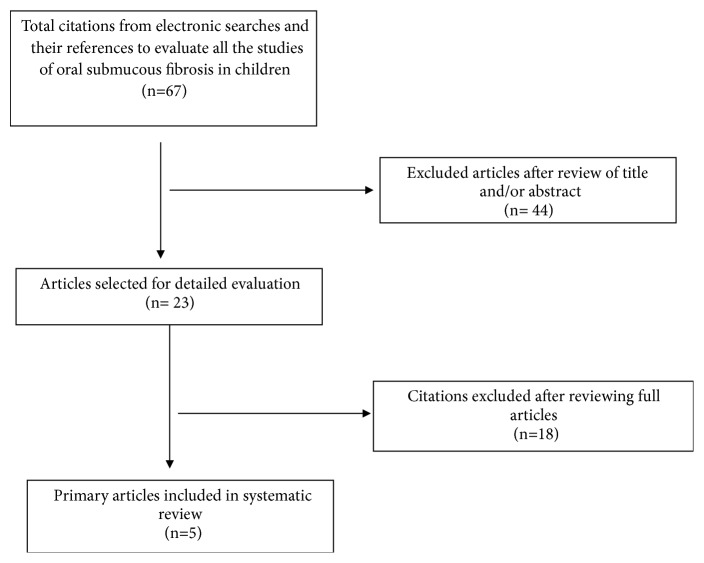
Flow diagram of study selection process.

The results of literature search are represented in Figure 1.

A total of sixty-seven articles were obtained after the initial search using keywords ‘oral submucous fibrosis' and ‘children'. After reviewing the abstracts and titles, twenty-three articles were selected. Out of these, only five articles were considered for inclusion in the systematic review and eighteen were excluded. The characteristics of included and excluded studies are depicted in Tables 1 and 2, respectively.

Data was then collected from five articles and a total of 10 cases of OSMF in pediatric patients were found. The youngest patient reported to be suffering from OSMF was of 2.5 years of age whereas the eldest was 10 years old. Considering the gender distribution female preponderance was noticed. Most of the cases were from Asian continent only. Etiology for occurrence of OSMF in all the included cases was areca nut chewing. Common site for placement of areca nut was buccal mucosa and others chewed it. Blanching of buccal mucosa, trismus, and burning sensation on eating were the symptoms noticed in almost all patients.

## 4. Discussion

OSMF is a premalignant condition which is more prevalent in Asian countries and Asians settled in other countries. Histologically, it is characterized by juxta-epithelial fibrosis with accumulation of hyalinized collagen with loss of vascularity and atrophy of epithelium [8]. Although the etiology of this condition is uncertain, there is enough epidemiological evidence proving areca nut chewing as the most common causative factor in its pathogenesis. Study by Harvey et al. on human fibroblasts proves that alkaloids present in areca nut cause OSMF by increasing collagen synthesis by 170% [8].

Three active ingredients present in areca nut are alkaloids, nitrosamines, and tannins. Alkaloids identified in areca nut by biochemical studies are arecoline, arecaidine, guvacine, and guvacoline. Arecoline is the main alkaloid which is carcinogenic [45]. These genotoxic substances affect the oral mucosal fibroblasts inducing fibroblast proliferation and collagen production [12]. There is progressive fibrosis of lamina propria and deeper connective tissues resulting in stiffening of oral mucosa and, thus, difficulty in opening of mouth. Chewing areca nut also produces cell mutagenicity and tumorigenicity [7, 46].

In the past few years the production and consumption of areca nut have increased in the country. Its sale is commercially aimed at children, and availability around school premises is making the young generations addicted to it [47, 48]. In the present study the pediatric patients affected by OSMF range from 2.5 to 10 years old. The younger the age of onset of habit, the more the chances of addiction and its consequences [49]. Most of the children and their families have no awareness about the harmful effects of areca nut. A study conducted by Oakley depicted that only 5% of children were aware of the malignant potential of areca nut [36]. Frequency of areca nut chewing is more common in children of low socioeconomic status because of low level of education and awareness among their families. Children also become habitual of areca nut chewing under peer pressure. In the present study, the gender wise distribution showed female predilection of OSMF. Areca nut chewing was more common among young girls probably because of the sweet taste, easy availability, and attractive packing. Therefore, lack of knowledge among parents, low cost, easy procurement, and widespread advertising are increasingly attracting children and making them addictive at an early age.

**Figure 2:**
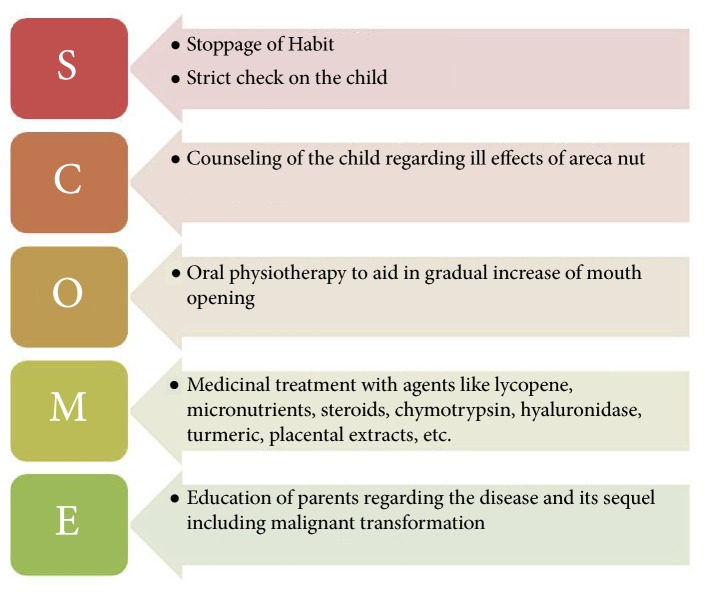
Protocol for management of Oral Submucous Fibrosis in Pediatric Patients.

Prevention is the best strategy to decrease the rate of OSMF cases affecting children. The biggest challenge is to motivate not only children but also their families not to indulge into areca nut chewing habits and to realize the associated health risks. As this dreadful condition is in a budding stage among this age group, a more focused but conservative approach is needed for management. Hence, authors have developed a protocol for management of OSMF in pediatric population (Figure 2). Steps should be taken at public health level to spread awareness through educational campaigns, newspapers, and mass media in communities and schools. A program for teacher training and education programs for children should be included in school curriculum; health professionals should also counsel and encourage the children for cessation of the habit. Policy makers should also come up with strategies like increasing taxes and not allowing sale of areca nut near schools to help children quit the habit.

## 5. Conclusion

Increasing cases of pediatric OSMF constitute a potential danger for the children in the society which is based on areca nut chewing habit. Lack of health consciousness and low level of education are the major factors for initiation of this habit among children. Early commencement of this habit leads to addiction and increased tendency for malignant transformation of OSMF. Therefore it is imperative for the parents and school as well as government authorities to take serious actions for cessation of the habit at primary level itself.

## Figures and Tables

**Table 1:** Characteristics of studies included in the systematic review.

S. No.	Author and Year	No. of Patients	Mean Age (years)	Gender	Habit
1.	R K Mundra et al. (1999) [22]	1	8	Female	Areca nut chewing

2.	Hazarey et al. (2007) [23]	1	4.5	Female	Areca nut chewing

3.	Sitheeque et al. (2010) [24]	5	3	3 Males	Areca nut chewing
			2.5	2 Females	Areca nut chewing

4.	Agrawal et al. (2011) [25]	1	9	Female	Areca nut chewing

5.	Dhariwal et al. (2012) [26]	2	10	Male	Areca nut chewing

**Table 2:** Characteristics of studies excluded from the systematic review.

S. No.	Author and Year	Reason for exclusion
1.	Yang et al. (2018) [27]	Complete data isn't available in the manuscript.

2.	Chitguppi et al. (2017) [28]	Number of cases in the 1^st^ decade not specified separately.

3.	Khandelwal et al. (2016) [29]	Patient was above 10 years.

4.	Singhvi et al. (2016) [30]	Number of cases in the 1^st^ decade not specified separately.

5.	Prasad et al. (2014) [31]	Pediatric OSMF cases are not mentioned separately in the study.

6.	Deshpande et al. (2013) [32]	Patient was above 10 years.

7.	Gupta et al. (2013) [33]	Patient was above 10 years.

8.	Khandelwal et al. (2012) [34]	No cases of pediatric OSMF are discussed in the study.

9.	Ahmad MS et al. (2006) [35]	All the patients were above 10 years.

10.	Eric Oakley et al. (2005) [36]	High school students were examined who were above 10 years.

11.	Rajendran R (2004) [37]	Doesn't include cases of pediatric OSMF.

12.	Shah et al. (2002) [38]	Characteristics of areca nut chewing in primary school children have been mentioned but not the prevalence of OSMF.

13.	Yusuf et al. (2002) [39]	Patient was above 10 years of age.

14.	Farrand et al. (2001) [40]	Consists of details on areca nut chewing habit in children but not about OSMF caused by it.

15.	Ranganathan et al. (2000) [41]	Just one case of OSMF in HIV patients is mentioned but age not specified.

16.	Shah et al. (2001) [42]	Patient was above 10 years of age.

17.	Lu et al. (1993) [43]	Discusses areca nut habit in children but not OSMF.

18.	Anil S et al. (1993) [44]	Patient was above 10 years of age.
